# Impact of Obesity, Smoking, and Alcohol on Erectile Dysfunction: A Cross-Sectional Study of Lebanese Men

**DOI:** 10.7759/cureus.95891

**Published:** 2025-11-01

**Authors:** Imad Ghantous, Mohamad Tlais, Ali Khatib, Georges Ghantous, Maria Sahyoun, Ali Hteit, Alaa Tarchichi, George Maroun, Nehme Jaber, Anthony Challita

**Affiliations:** 1 Urology, Saint George University Hospital, Beirut, LBN; 2 Faculty of Medicine and Medical Sciences, University of Balamand, Beirut, LBN; 3 Urology, Saint George University Medical Center, Beirut, LBN

**Keywords:** age, alcohol, erectile dysfunction, obesity, smoking

## Abstract

Erectile dysfunction (ED) is a significant health issue affecting men globally, with established risk factors including age, smoking, obesity, and alcohol consumption. Despite its widespread prevalence, limited data exist on ED and its associated risk factors within the Lebanese population. This study aimed to assess the prevalence and severity of ED among Lebanese men aged 45 and older and examine its relationship with lifestyle factors such as smoking, obesity, and alcohol consumption. A total of 150 participants were recruited during prostate health awareness campaigns, and ED was evaluated using the International Index of Erectile Function (IIEF-5). Data on body mass index (BMI), smoking status, and alcohol consumption were collected through structured questionnaires. Statistical analyses, including chi-square tests and logistic regression, were conducted to explore associations between these factors and ED severity. The findings revealed a high prevalence of ED, with 130 (86.7%) of participants reporting varying degrees of dysfunction. BMI and smoking were significantly associated with increased ED severity (p < 0.01), while age and alcohol consumption were not significant predictors. Logistic regression analysis identified BMI as the strongest predictor of severe ED (OR = 1.21, p = 0.005). These results underscore the critical roles of smoking and obesity in the progression of ED among Lebanese men and highlight the need for public health interventions focused on weight management and smoking cessation. This study provides valuable localized insights into ED risk factors, offering opportunities for preventive strategies tailored to this population.

## Introduction

Erectile dysfunction (ED) is one of the most prevalent conditions affecting males worldwide. The European Association of Urology (EAU) defines ED as “the inability to attain and maintain an erection sufficient to perform satisfactory sexual intercourse” [1]. ED is more common in older men with approximately 52% of men worldwide aged from 40 to 70 years suffering from varying degrees of ED, with prevalence increasing with age [2]. Left untreated, ED can have adverse effects on the quality of life of both the men affected by ED and their female partners [3]. Additionally, psychosocial problems (i.e., anxiety and depression), withdrawal from sexual intimacy, and even decreased productivity at work can all occur as a result of ED [4,5].

There are various risk factors associated with ED, with the most prominent ones being age, smoking, obesity, and alcohol consumption [6,7]. Also, ED is prevalent in patients with comorbidities like cardiovascular disease and diabetes mellites [8,9]. Additionally, mental health conditions such as depression and anxiety commonly feature ED [10,11]. ED is unanimously considered multifactorial in etiology [12]. The two non-mutually exclusive causes are organic and psychological. Broadly, organic causes can further be divided into endocrine and non-endocrine. Non-endocrine causes could be vascular, neurologic, or iatrogenic. In contrast, endocrine causes are anything that can lead to a lower serum testosterone level [13].

The current lack of data on ED prevalence and risk factors across the Levant region makes studying this population particularly significant. The distinctive sociocultural and environmental factors of Lebanon, such as the Mediterranean diet, the widespread use of tobacco, and the varied levels of alcohol consumption across different religions and ethnicities, all provide a unique context for examining ED. In particular, cultural norms that discourage open discussion of sexual health, the social acceptance of smoking (including waterpipe use), and dietary and lifestyle habits shaped by religious and socioeconomic diversity may all modulate ED risk. Additionally, analyzing the interactions of these different risk factors in ED may present differently from previously studied populations; thus, it is important to understand the contribution of these factors to the severity and prevalence of ED in the Lebanese population.

With that said, the specific cultural, lifestyle, and genetic factors offer a unique risk profile for Lebanon. This risk profile could add crucial insight to the ED risk factors that are already established in the literature. Hence, our study aims to fill the gap by presenting localized data on the prevalence of ED and its risk factors in Lebanon.

Understanding and identifying risk factors such as age, smoking, BMI, and alcohol enables the early detection of ED by healthcare providers. Early detection coupled with timely intervention can hinder any further progression of ED [1]. Furthermore, by identifying risk factors, personalized treatment plans can be developed, which leads to improved patient outcomes. For instance, interventions such as smoking cessation and/or weight loss programs can be incorporated into the treatment regimen for patients based on their lifestyle.

Risk factors

Age has been well-established in the literature as a significant risk factor for ED [14-18]. These studies reported the increased prevalence of ED with advancing age using broad categories like 10-year age intervals. For instance, regular sexual activity in Braun et al.’s study [16] was 96.0% (30-40 age group) to 71.3% (70-80 age group). Similarly, Mulhall et al. [17] found: “Prevalence of ED diagnosis or treatment increased from age 18-29 years (0.4%) to 60-69 years (11.5%)”. This approach has been criticized by Pelligrino et al. [14], who considered it inaccurate due to the variations that could exist between the age gaps. For example, a 60-year-old may not have the same ED risk score as a 69-year-old, but they are regarded as the same age group nonetheless. These studies also used mixed populations with various comorbidities that are well-known risk factors for ED such as diabetes mellitus, hypertension, and obesity [19]. To address this, Pelligrino et al. [14] investigated the risk of ED in the context of age alone and accounted for any existing comorbidities. They found that the prevalence of patient-reported ED increased from 10% to 79% in the 40 to 80-year age groups, respectively, in men without any comorbidities. Meanwhile, patients with comorbidities showed a sharp increase in ED risk. Specifically, the probability of ED for 50- and 75-year-old individuals was 20% and 68% in men with no comorbidities; while those with hypertension, diabetes, and obesity had 41% and 85% ED probabilities, respectively. The effect of age on ED can be attributed to the decreased vascular health associated with aging, specifically the dysfunction of the corporal smooth muscle cells [20]. The decrease in testosterone level with age is a significant cause of ED as well [21].

Various studies have highlighted the role of smoking as a risk factor for ED [22,23]. Significantly, the meta-analysis conducted by Cao et al. [6], which included over 28 thousand participants distributed over four cohorts and four case-control studies, found smoking to be an important risk factor for ED. This occurs due to the debilitating effects of smoking on the vascular endothelium and peripheral nerves as well as damage to the corporal tissue of the penis [23].

Obesity has long been known as a major risk factor for ED [24]. In their review, Moon et al. [25] established that obesity, through its adverse effects on endothelial function [26], its subsequent insulin and leptin resistance [27], and its lowering of testosterone levels, will eventually result in ED.

There are mixed results regarding the effects of alcohol consumption on ED. Some animal studies found that chronic consumption of high doses of alcohol for six weeks contributes to ED through increasing the production of reactive oxygen species in the corpus cavernosum [28-30]. Likewise, studies on the general human population also found that chronic consumption of high alcohol doses has adverse effects on erectile function [21,22]. Meanwhile, other studies found a protective effect on ED in light-to-moderate consumption of alcohol [23,24]. The protective effects of alcohol on ED involve both biological mechanisms and psychological factors. Biologically, alcohol exhibits long-term benefits on high-density lipoprotein (HDL) cholesterol, which, in turn, increases the activity and availability of nitric oxide necessary to maintain an erection. Moreover, consuming light-to-moderate amounts of alcohol boosts the expression and activity of paraoxonase-1 in the bloodstream. In contrast, heavy drinking reduces these levels in both animals and humans. Paraoxonase-1 plays a role in neutralizing homocysteine, thus preventing its potential harmful impact on the endothelium [25]. Psychologically, alcohol is known to play a key role in reducing sexual performance anxiety and promote relaxation and disinhibition, all of which are beneficial for erectile function prior to intercourse [26]. This scarcity of research likely reflects cultural sensitivities surrounding sexual health discussions and underreporting of ED within the Lebanese and broader Levantine context.

Objective

This study aims to identify the prevalence of ED in the understudied Lebanese population; examine the relationship between age and ED; as well as analyze the associations of lifestyle factors such as smoking, drinking, and BMI with ED. Moreover, this study will compare the severity and prevalence of ED in different lifestyle groups. This will allow us to comprehensively analyze the contribution of these factors to ED in the Lebanese population with its mixed cultural background and geographic distribution.

## Materials and methods

Study design

This study was a cross-sectional observational analysis conducted to investigate the prevalence of ED and its association with risk factors such as age, smoking, alcohol consumption, and obesity (BMI) among adult males in Lebanon. The study was carried out as part of a men's health awareness campaign focusing on prostate health, targeting males over the age of 45. Data were collected during prostate health screening events held in Saint George Hospital University Medical Center in Beirut as part of a community awareness campaign. No clinical or biochemical verification of erectile function was performed; data relied entirely on self-reported information.

Study population

Participants in this study were adult males aged 45 years and older who attended prostate health campaign screenings. The inclusion criteria required participants to be males aged 45 and above who provided informed consent and were willing to complete the questionnaire. Participants under the age of 45 and those unable to complete the questionnaire independently were excluded from the study.

Sample size

A total of 150 participants were included in the study, representing the number of respondents during the campaign. This sample size was considered sufficient based on power calculations, allowing for the detection of significant differences in ED prevalence across various risk factors (age, smoking status, alcohol consumption, and BMI) with 80% power and a 5% significance level.

Data collection tools

Data were collected through a structured, self-administered questionnaire that covered multiple sections. The demographic information section collected details on participants’ age, marital status, educational level, and geographic region. The medical history section gathered information on comorbidities such as hypertension, diabetes, cardiovascular disease, and current medications. The lifestyle factors section assessed smoking status, categorized as current smoker, past smoker, or never smoked; alcohol consumption, categorized as light, moderate, or heavy drinking based on frequency and amount; and body mass index (BMI), calculated from self-reported height and weight, categorized into normal, overweight, or obese according to World Health Organization (WHO) standards. Alcohol consumption was categorized as light (≤1 drink/week), moderate (2-7 drinks/week), or heavy (>7 drinks/week), following WHO guidelines for population alcohol surveys. Erectile dysfunction was assessed using the International Index of Erectile Function (IIEF-5), which categorizes ED severity into no ED, mild, moderate, or severe.

Data analysis

Data analysis was performed using statistical software SPSS to conduct both descriptive and inferential analysis. Descriptive statistics were calculated to summarize the sample’s demographics and lifestyle characteristics, including means, frequencies, and percentages. The overall prevalence of ED in the study sample was calculated, as well as the prevalence within each risk factor category (age, smoking status, alcohol consumption, and BMI). A chi-square test was used to assess associations between categorical variables, while logistic regression was employed to evaluate the strength of association between ED and the main risk factors (age, BMI, smoking, and alcohol consumption), with odds ratios (OR) and 95% confidence intervals (CI) reported. Additionally, stratified subgroup analyses were conducted to explore differences in ED prevalence and severity across various geographic regions and socioeconomic groups within the Lebanese population.

Ethical considerations

Consent was obtained or waived by all participants in this study. Institutional Review Board (IRB) of Saint George Hospital University Medical Center, Beirut, Lebanon issued approval FWA00015224. This study was conducted in accordance with the principles of the Declaration of Helsinki. All participants provided written informed consent prior to their inclusion in the study.

Data availability

The data collected during this study are securely stored and available upon request for future research purposes, subject to IRB approval.

## Results

Participant demographics

The study analyzed data from 150 Lebanese male participants aged 45 and above. The mean age of the participants was 63.15 years (SD = 7.16), with a range from 50 to 80 years. The cohort predominantly consisted of married individuals, accounting for 128 participants (85%) of the sample, while the remaining 22 participants (15%) were single (Table 1).

**Table 1:** Demographic characteristics of study participants

Characteristic	Value
Total participants	150
Mean age (SD)	63.15 (7.16) years
Age range	50–80 years
Marital status: Married	128 (85%)
Marital status: Single	22 (15%)

Prevalence of ED

Based on the International Index of Erectile Function (IIEF-5) scoring, the severity of ED among participants was distributed as follows: 50 participants (33.3%) experienced mild-to-moderate ED, 33 participants (22.0%) had mild ED, 29 participants (19.3%) experienced moderate ED, and 18 participants (12.0%) reported severe ED. Meanwhile, 20 participants (13.3%) reported no ED. These findings indicate that a substantial proportion of the sample experienced varying degrees of ED, with more severe forms being less prevalent (Table 2).

**Table 2:** Distribution of erectile dysfunction severity among participants ED: Erectile dysfunction.

ED severity	N (%)
No ED	20 (13.3%)
Mild ED	33 (22.0%)
Moderate ED	29 (19.3%)
Mild-to-moderate ED	50 (33.3%)
Severe ED	18 (12.0%)

Association between ED and risk factors

The analysis of age and ED severity revealed no statistically significant association, with a chi-square value of 12.42 and a p-value of 0.41, indicating that although the prevalence of ED increased with age, it did not independently predict ED severity within this sample. In contrast, the relationship between BMI and ED severity was significant, as evidenced by a chi-square value of 29.72 and a p-value of 0.003. Participants with normal BMI were primarily associated with mild and mild-to-moderate ED, whereas those in the overweight and obese categories showed higher occurrences of moderate and severe ED. Cases of severe ED were also identified among participants classified as severely obese, although their numbers were fewer. Smoking was another significant factor, with a chi-square value of 22.78 and a p-value of 0.00014, demonstrating that smokers were more likely to experience severe ED compared to nonsmokers, emphasizing smoking as a critical contributor to ED severity in this population. On the other hand, the relationship between alcohol consumption and ED severity was not statistically significant, with a chi-square value of 13.57 and a p-value of 0.33. While some variations were observed, no strong correlation was established between alcohol consumption levels and ED severity in this sample (Table 3).

**Table 3:** Association between risk factors and erectile dysfunction severity

Risk factor	Chi-square value	p-value	Significance
Age	12.42	0.41	Not significant
BMI	29.72	0.003	Significant
Smoking	22.78	0.00014	Significant
Alcohol consumption	13.57	0.33	Not significant

Logistic regression analysis

A logistic regression model was employed to further examine the impact of age, BMI, alcohol consumption, and smoking status on severe ED. Age had an OR of 0.99 (p = 0.828), showing no significant effect. BMI was a significant predictor, with an OR of 1.21 (p = 0.005). Alcohol consumption showed a nonsignificant inverse relationship, with an OR of 0.91 (p = 0.111). Although the chi-square test highlighted the significance of smoking, the regression analysis suggested that BMI had a more pronounced effect when combined with other factors (Table 4).

**Table 4:** Logistic regression analysis of predictors of severe erectile dysfunction

Predictor	Odds ratio (OR)	p-value	Interpretation
Age	0.99	0.828	No significant effect
BMI	1.21	0.005	Significant predictor
Smoking	—	0.00014	Significant in the Chi-square test
Alcohol consumption	0.91	0.111	No significant effect

Model performance

The model demonstrated moderate fit, with a pseudo R-squared value of 0.1085. The likelihood ratio test confirmed the overall model's significance (p-value = 0.0078), indicating that the included risk factors contributed to predicting severe ED.

The results of this study demonstrate that BMI and smoking are significantly associated with ED severity among Lebanese men aged 45 and older. While age and alcohol consumption were not significant independent predictors, smoking showed a marked impact on ED severity. Public health measures focusing on weight management and smoking cessation could play a pivotal role in mitigating ED risk in this population. Further research should explore additional risk factors and involve larger samples to enhance the generalizability of these findings.

## Discussion

This study underscores the prevalence and severity of ED among Lebanese men aged 45 and older, revealing that a significant portion of the cohort experienced some form of ED. Specifically, 130 participants (86.7%) displayed varying degrees of ED, ranging from mild to severe, while 20 participants (13.3%) reported no ED. The most common category was mild-to-moderate ED, affecting 50 participants (33.3%), whereas severe ED was the least frequent, occurring in 18 participants (12.0%) of the cohort.

The association analysis provided critical insights into the relationship between ED severity and various risk factors. A significant relationship was found between BMI categories and ED severity, with a chi-square value of 29.72 and a p-value of 0.003. Participants with higher BMI values-those classified as overweight, obese, or severely obese-were more likely to report moderate to severe ED. This underscores the role of weight as a major determinant of ED severity. In the logistic regression model, BMI remained a significant predictor of severe ED, with an OR of 1.21 (p = 0.005).

Smoking was another significant factor linked to ED severity. The chi-square analysis yielded a value of 22.78 with a p-value of 0.00014, demonstrating a strong correlation. Smokers exhibited a higher likelihood of severe ED compared to nonsmokers, emphasizing smoking as a critical risk factor. However, in the logistic regression model, BMI had a more pronounced effect on ED severity than smoking when combined with other factors.

In contrast, age and alcohol consumption showed no significant associations with ED severity in this sample. Although the prevalence of ED increased with age, the chi-square test indicated no statistically significant relationship between age groups and ED severity (χ² = 12.42, p = 0.41). The OR for age in predicting severe ED was 0.99 (p = 0.828), further supporting the lack of significance. Similarly, alcohol consumption did not exhibit a meaningful association with ED severity, as indicated by a chi-square value of 13.57 and a p-value of 0.33. The odds ratio for alcohol consumption suggested a nonsignificant inverse relationship (OR = 0.91, p = 0.111).

These results highlight BMI and smoking as significant risk factors for ED severity among this population. On the other hand, age and alcohol consumption did not independently predict ED severity. These findings emphasize the importance of addressing modifiable risk factors such as weight management and smoking cessation to mitigate the risk of ED.

Interpretations

The correlation found in this study between higher BMI values and severe ED is consistent with existing literature [24]. Obesity is known to deteriorate erectile function through its adverse effects on vasculature and testosterone levels [25]. Smoking as a risk factor has been reinforced once again in our study as a risk factor for ED, similar to past literature. The adverse effects of smoking on erectile function could be attributed to endothelial dysfunction and reduced blood flow [22,23]. While ED prevalence increased with age, the association between age and ED severity was not found in this study. This contradicts current literature that affirms age as a major risk factor for ED [14-18]. This contradiction indicates the priority of lifestyle factors (i.e., diet and smoking) in predicting ED over factors like age in our specific Lebanese population. Meanwhile, the lack of association between alcohol and ED in our study reflected the mixed findings in the literature. Some studies found alcohol to be a protective factor [23,24], while others found its chronic use to be a risk factor [28-30]. The lack of significant correlations could be attributed to Lebanon's precarious drinking culture, where alcohol consumption is frowned upon in some communities while considered permissible in others.

The findings of this study can be contextualized within the unique cultural and dietary practices of Lebanon, which may influence ED risk factors. The Mediterranean diet, commonly practiced in Lebanon, is associated with cardiovascular benefits that could reduce ED risk. However, its protective effects may be offset by the high prevalence of smoking, including waterpipe (argileh) smoking, which is a socially and culturally accepted practice.

In addition, the patterns of alcohol consumption in Lebanon vary greatly across religious and social groups, complicating the analysis of its relationship with ED. While light-to-moderate alcohol consumption is known to have potential protective effects, heavy drinking may counteract these benefits. This variability in drinking habits likely contributed to the lack of a significant association between alcohol consumption and ED severity in our sample.

Generalizability

It is worth noting that the results may not apply to the general population due to the unique culture and demographics of Lebanon. Limited generalizability may also apply in Lebanon itself due to the various ethnic, religious, and cultural differences in the relatively small country. Therefore, repeating the study in more peripheral areas of Lebanon could yield different results. Additionally, since the sample comprises only men aged 45 and above, these results may not be representative of younger age groups. Younger age groups might have different risk factors or lifestyle behaviors that influence ED prevalence and severity. Future studies in other Lebanese regions and demographic groups are required to enhance representativeness and applicability to the wider population.

Limitations

This study has several limitations that should be acknowledged. First, its cross-sectional design precludes the establishment of causal relationships between risk factors and ED; the associations identified cannot be interpreted as evidence of causality. Second, the study relied entirely on self-reported questionnaire data without clinical examination or laboratory confirmation of comorbidities or erectile function, which may have introduced recall and reporting bias.

Third, the sampling method was based on convenience sampling of participants attending prostate health awareness campaigns held in hospitals and community health centers. As a result, the findings may not be fully representative of the broader Lebanese male population, limiting external validity and generalizability. Additionally, the sample size was modest (n = 150), which may have reduced the statistical power to detect smaller associations and may not reflect the diversity of Lebanon’s demographic and cultural subgroups.

Fourth, while information on comorbidities such as hypertension, diabetes mellitus, and cardiovascular disease was collected, it was not included in the multivariable regression analysis due to incomplete reporting. This omission may have led to residual confounding, particularly given the established influence of these conditions on erectile function. Moreover, important behavioral details such as the intensity and duration of smoking (e.g., pack-years) and the exact quantity of alcohol intake were not quantified beyond categorical classification, limiting the precision of exposure measurement.

Finally, no validated screening tool such as the AUDIT-C was used to categorize alcohol consumption, and no longitudinal follow-up was performed to assess changes in erectile function over time. Future research should incorporate larger, population-based samples, objective clinical and biochemical evaluations, and a comprehensive assessment of lifestyle and psychosocial variables to better clarify causal pathways and enhance generalizability.

## Conclusions

This study highlights the high prevalence of ED among Lebanese men aged 45 and above, with lifestyle factors such as obesity and smoking playing a pivotal role in determining ED severity. Specifically, higher BMI and smoking were significantly associated with moderate to severe ED, emphasizing the detrimental impact of these modifiable risk factors. Conversely, no significant association was found between alcohol consumption or age and ED severity, suggesting that lifestyle factors showed stronger associations with ED severity than age within this study’s sample.

These findings carry important implications for public health strategies in Lebanon, where targeted interventions such as smoking cessation programs and weight management initiatives could mitigate ED risk and improve overall sexual health. Future studies should include a more diverse demographic and larger sample sizes to validate these findings and explore additional contributing factors such as psychological health and comorbidities. Expanding research to include younger populations and the role of cultural and socioeconomic influences on ED could further enrich the understanding of this condition in Lebanon.

## Appendices

Erectile dysfunction study questionnaire

The questionnaire used in the study to collect demographic, medical, lifestyle, and erectile dysfunction-related data is presented in Table 5.

**Table 5:** Questionnaire used for data collection

Section	Question	Response Option
Demographic Information	Age	Open-ended
Demographic Information	Marital Status	Married
Demographic Information	Marital Status	Single
Demographic Information	Marital Status	Divorced
Demographic Information	Marital Status	Widowed
Demographic Information	Educational Level	Primary
Demographic Information	Educational Level	Secondary
Demographic Information	Educational Level	Tertiary
Demographic Information	Geographic Region	Open-ended
Medical History	Do you have any of the following conditions?	Hypertension
Medical History	Do you have any of the following conditions?	Diabetes Mellitus
Medical History	Do you have any of the following conditions?	Cardiovascular Disease
Medical History	Do you have any of the following conditions?	Other (Specify)
Medical History	Are you currently taking any medication?	Yes (Specify)
Medical History	Are you currently taking any medication?	No
Lifestyle Factors	Smoking Status	Current Smoker
Lifestyle Factors	Smoking Status	Past Smoker
Lifestyle Factors	Smoking Status	Never Smoked
Lifestyle Factors	Alcohol Consumption	Light Drinker
Lifestyle Factors	Alcohol Consumption	Moderate Drinker
Lifestyle Factors	Alcohol Consumption	Heavy Drinker
Lifestyle Factors	Body Mass Index (BMI) - Height (cm)	Open-ended
Lifestyle Factors	Body Mass Index (BMI) - Weight (kg)	Open-ended
Erectile Dysfunction Assessment (IIEF-5)	How do you rate your confidence that you could get and keep an erection?	1 = Very low
Erectile Dysfunction Assessment (IIEF-5)	How do you rate your confidence that you could get and keep an erection?	2 = Low
Erectile Dysfunction Assessment (IIEF-5)	How do you rate your confidence that you could get and keep an erection?	3 = Moderate
Erectile Dysfunction Assessment (IIEF-5)	How do you rate your confidence that you could get and keep an erection?	4 = High
Erectile Dysfunction Assessment (IIEF-5)	How do you rate your confidence that you could get and keep an erection?	5 = Very high
Erectile Dysfunction Assessment (IIEF-5)	When you had erections with sexual stimulation, how often were your erections hard enough for penetration?	1 = Almost never
Erectile Dysfunction Assessment (IIEF-5)	When you had erections with sexual stimulation, how often were your erections hard enough for penetration?	2 = A few times
Erectile Dysfunction Assessment (IIEF-5)	When you had erections with sexual stimulation, how often were your erections hard enough for penetration?	3 = Sometimes
Erectile Dysfunction Assessment (IIEF-5)	When you had erections with sexual stimulation, how often were your erections hard enough for penetration?	4 = Most times
Erectile Dysfunction Assessment (IIEF-5)	When you had erections with sexual stimulation, how often were your erections hard enough for penetration?	5 = Almost always
Erectile Dysfunction Assessment (IIEF-5)	During sexual intercourse, how often were you able to maintain your erection after penetration?	1 = Almost never
Erectile Dysfunction Assessment (IIEF-5)	During sexual intercourse, how often were you able to maintain your erection after penetration?	2 = A few times
Erectile Dysfunction Assessment (IIEF-5)	During sexual intercourse, how often were you able to maintain your erection after penetration?	3 = Sometimes
Erectile Dysfunction Assessment (IIEF-5)	During sexual intercourse, how often were you able to maintain your erection after penetration?	4 = Most times
Erectile Dysfunction Assessment (IIEF-5)	During sexual intercourse, how often were you able to maintain your erection after penetration?	5 = Almost always
Erectile Dysfunction Assessment (IIEF-5)	During sexual intercourse, how difficult was it to maintain your erection to completion of intercourse?	1 = Extremely difficult
Erectile Dysfunction Assessment (IIEF-5)	During sexual intercourse, how difficult was it to maintain your erection to completion of intercourse?	2 = Very difficult
Erectile Dysfunction Assessment (IIEF-5)	During sexual intercourse, how difficult was it to maintain your erection to completion of intercourse?	3 = Difficult
Erectile Dysfunction Assessment (IIEF-5)	During sexual intercourse, how difficult was it to maintain your erection to completion of intercourse?	4 = Slightly difficult
Erectile Dysfunction Assessment (IIEF-5)	During sexual intercourse, how difficult was it to maintain your erection to completion of intercourse?	5 = Not difficult
Erectile Dysfunction Assessment (IIEF-5)	When you attempted sexual intercourse, how often was it satisfactory for you?	1 = Almost never
Erectile Dysfunction Assessment (IIEF-5)	When you attempted sexual intercourse, how often was it satisfactory for you?	2 = A few times
Erectile Dysfunction Assessment (IIEF-5)	When you attempted sexual intercourse, how often was it satisfactory for you?	3 = Sometimes
Erectile Dysfunction Assessment (IIEF-5)	When you attempted sexual intercourse, how often was it satisfactory for you?	4 = Most times
Erectile Dysfunction Assessment (IIEF-5)	When you attempted sexual intercourse, how often was it satisfactory for you?	5 = Almost always
Additional Comments	Please provide any additional comments related to your sexual health or lifestyle factors.	Open-ended
